# Effect of skin–capsular distance on controlled attenuation parameter for diagnosing liver steatosis in patients with nonalcoholic fatty liver disease

**DOI:** 10.1038/s41598-021-94970-3

**Published:** 2021-08-02

**Authors:** Syunichiro Kimura, Kenichi Tanaka, Satoshi Oeda, Kaori Inoue, Chika Inadomi, Yoshihito Kubotsu, Wataru Yoshioka, Michiaki Okada, Hiroshi Isoda, Takuya Kuwashiro, Takumi Akiyama, Aya Kurashige, Ayaka Oshima, Mayumi Oshima, Yasue Matsumoto, Atsushi Kawaguchi, Keizo Anzai, Eisaburo Sueoka, Shinichi Aishima, Hirokazu Takahashi

**Affiliations:** 1 Division of Metabolism and Endocrinology, Faculty of Medicine, Saga University, 5-1-1 Nabeshima, Saga, 849-8501 Japan; 2 Liver Center, Saga University Hospital, 5-1-1 Nabeshima, Saga, 849-8501 Japan; 3 Department of Laboratory Medicine, Saga University Hospital, 5-1-1 Nabeshima, Saga, 849-8501 Japan; 4 Education and Research Center for Community Medicine, Faculty of Medicine, Saga University, 5-1-1 Nabeshima, Saga, 849-8501 Japan; 5 Department of Clinical Laboratory Medicine, Faculty of Medicine, Saga University, 5-1-1 Nabeshima, Saga, 849-8501 Japan; 6 Department of Pathology & Microbiology, Faculty of Medicine, Saga University, 5-1-1 Nabeshima, Saga, 849-8501 Japan

**Keywords:** Hepatology, Gastrointestinal diseases

## Abstract

The effect of the skin–capsular distance (SCD) on the controlled attenuation parameter (CAP) for diagnosis of liver steatosis in patients with nonalcoholic fatty liver disease (NAFLD) remains unclear. The SCD was measured using B-mode ultrasound, and the CAP was measured using the M probe of FibroScan^®^. According to the indications of the M probe, 113 patients with an SCD of ≤ 25 mm were included in the present study. The association between the SCD and CAP was investigated, and the diagnostic performance of the SCD-adjusted CAP was tested. The SCD showed the most significant positive correlation with the CAP (ρ = 0.329, p < 0.001). In the multiple regression analysis, the SCD and serum albumin concentration were associated with the CAP, independent of pathological liver steatosis. According to the multivariate analysis, two different formulas were developed to obtain the adjusted CAP using the SCD and serum albumin concentration as follows: adjusted CAP (dB/m) = CAP − (5.26 × SCD) and adjusted CAP (dB/m) = CAP − (5.35 × SCD) − (25.77 × serum albumin concentration). The area under the receiver operating characteristic curve for diagnosis of a steatosis score ≥ 2 of adjusted CAP was 0.678 and 0.684 respectively, which were significantly greater than the original CAP (0.621: p = 0.030 and p = 0.024). The SCD is associated with the CAP independent of liver steatosis. Adjustment of the CAP using the SCD improves the diagnostic performance of the CAP in NAFLD.

## Introduction

Nonalcoholic fatty liver disease (NAFLD) is a chronic liver disease caused by obesity and metabolic syndrome^1^. The presence of liver steatosis is the principal criterion for the diagnosis of NAFLD. Pathologically, a ≥ 5% area of steatosis in the liver parenchyma is required for the diagnosis of NAFLD^2^. Ultrasound is commonly used to detect liver steatosis, especially in the primary care setting. Liver steatosis is diagnosed by the finding of a “bright liver,” which is characterized by increased hepatic echogenicity, poor penetration of the posterior segment of the right lobe, and poor or no visualization of the hepatic vessels and diaphragm^3,4^. Hepatorenal echo contrast is a well-validated ultrasonographic finding of fatty liver; a ≥ 20% area of steatosis in a liver specimen has a sensitivity of 96.4% and specificity of 97.8% for the diagnosis of fatty liver^5^. However, subjective diagnosis of liver steatosis based on B-mode imaging shows wide interobserver and intraobserver variability^6^. Because of the global high prevalence of NAFLD and limitations of liver biopsy, including high invasiveness, high cost, and diagnostic discordance among pathologists^7–9^, a simple, reliable, and quantitative screening procedure to identify liver steatosis is warranted.

The FibroScan^®^ (Echosens, Paris, France) is an ultrasound-based transient elastography device that enables noninvasive evaluation of liver fibrosis through assessment of the liver stiffness measurement (LSM) and steatosis through assessment of the controlled attenuation parameter (CAP)^10,11^. The CAP represents the attenuation of the ultrasound beam in the liver. More severe liver steatosis produces a more attenuated ultrasound pulse passing through the liver tissue. The CAP value ranges from 100 to 400 dB/m based on this property^12^. CAP measurement is useful for diagnosing pathological liver steatosis in patients with NAFLD^11,13–15^. Moreover, Caussy et al.^15^ reported that the CAP showed representable diagnostic accuracy when the proton density fat fraction measured by magnetic resonance imaging (MRI-PDFF) was used as a gold standard.

In terms of the clinical implications, it is important to know that the LSM and CAP are affected by several factors in addition to fibrosis and steatosis, respectively^16^. The LSM is increased by inflammation, venous pressure, cholestasis, and amyloid deposition in the liver^16–22^. Additionally, the body mass index (BMI) is reportedly a significant confounding factor that increases the CAP^11,16,23–28^. A greater distance between the skin surface and the liver capsule (Supplementary Figure 1), termed the skin–capsular distance (SCD), increases the LSM because of multiple ultrasonic echo reflections. In one study, the LSM increased in patients with an SCD of ≥ 20 mm^29^. However, whether the SCD affects the CAP remains unclear. The SCD includes the thickness of the subcutaneous fat tissue. Therefore, we hypothesized that the SCD affects the CAP of obese patients with NALFD and that the diagnostic performance of CAP measurement improves by adjustment of the SCD. This study was performed to clarify whether the SCD affects the CAP and to generate a formula for calculation of the adjusted CAP.

## Methods

### Patients

We retrospectively enrolled 150 consecutive patients who visited Saga University Hospital from May 2017 to August 2019. All patients were diagnosed with NAFLD by liver biopsy, and underwent CAP measurement with the M probe or XL probe of the FibroScan^®^. According to the indications of the M probe (SCD of ≤ 25 mm), 37 patients with an SCD of > 25 mm were excluded and the data of 113 patients were finally analyzed in the current study.

### Physical examination and serum biochemical measurements

Patients were diagnosed with diabetes mellitus if their fasting plasma glucose (FPG) concentration was > 126 mg/dL and/or they were undergoing treatment with antidiabetic drugs. Patients were diagnosed with hypertension if their systolic blood pressure was > 140 mmHg and/or diastolic blood pressure was > 90 mmHg and/or they were undergoing treatment with antihypertensive drugs. The patients’ body mass and height were measured, and the BMI was calculated as body mass (kg) divided by height squared (m^2^). Venous blood samples were obtained after overnight fasting and used to determine the following parameters by conventional laboratory techniques: blood cell counts, platelet count, FPG, and serum levels of aspartate aminotransferase, alanine transaminase, alkaline phosphatase, γ-glutamyl transpeptidase, total bilirubin, total protein, albumin, total cholesterol, triglycerides, high-density lipoprotein cholesterol, low-density lipoprotein cholesterol, ferritin, and type IV collagen 7 s.

### Liver biopsy and histological assessment

Ultrasonography-guided liver biopsy was performed using a 16-gauge biopsy needle. All liver biopsy specimens were approximately ≥ 20 mm in length. Liver biopsy slides stained with hematoxylin–eosin and Azan stain were evaluated by a single experienced pathologist (S.A.) specializing in liver pathology. The pathologist was blinded to the clinical data. NAFLD was pathologically diagnosed if the steatosis area was > 5%. Hepatic steatosis, lobular inflammation, and hepatocyte ballooning were evaluated using the NAFLD activity score^30^. Liver fibrosis was classified according to Kleiner et al.^30^ and Brunt et al.^31^. Steatosis was assigned a score of 0, 1, 2, or 3 (score of 0, < 5%; score of 1, 5–33%; score of 2, 34–66%; and score of 3, > 66% of biopsy specimen affected), and fibrosis was assigned a score of 0, 1, 2, 3, or 4 (stage 0, no fibrosis; stage 1, perisinusoidal or periportal fibrosis; stage 2, perisinusoidal and portal/periportal fibrosis; stage 3, bridging fibrosis; and stage 4, cirrhosis).

### LSM and CAP

Experienced operators who had performed at least 500 examinations assessed the CAP in the right liver lobe using the FibroScan^®^ 502. Patients were examined after an overnight fast using the M probe or XL probe within 6 months before and after liver biopsy. First, we observed the optimal site for FibroScan^®^ examination without blood vessels or any space-occupying lesions using B-mode ultrasound, and we measured the SCD. CAP measurements were performed using the FibroScan^®^ M probe for patients with an SCD of ≤ 25 mm and the XL probe for patients with an SCD of > 25 mm and ≤ 35 mm until 10 valid measurements were obtained for each patient, and the median values were used to quantify liver steatosis. CAP measurement was expressed in dB/m. Based on previous reports, we defined measurement failure as examinations in which 10 valid CAP measurement were not obtained after 10 or more acquisitions^32^.

### Statistical analysis

Correlations were tested using Spearman’s rank correlation coefficient. Single regression analysis and multiple regression analysis were performed to identify the factors associated with the CAP. Explanatory variables for the multiple regression analysis were chosen as follows: Model 1, pathological steatosis and CAP; Model 2, variables of Model 1 + BMI; Model 3, variables of Model 2 + significant variables in the single regression analysis. Using the partial regression coefficient (B) obtained in the multiple regression analysis (Models 1 and 3), the adjusted CAP was calculated as follows: Model 1-adjusted CAP = CAP − (B × SCD); Model 3-adjusted CAP (dB/m) = CAP − (B × SCD) − (B × serum albumin concentration). The diagnostic performance of the LSM and CAP measurements was determined using receiver operating characteristic (ROC) curves. The optimal cut-off values were chosen to maximize the sum of the sensitivity and specificity in Youden’s index^33^. Comparisons of the area under the ROC curve (AUROC) between the original CAP and adjusted CAP were performed using the DeLong test^34^. A p value of < 0.05 was considered statistically significant. All statistical analyses were conducted under the supervision of a statistical expert (A.K.), and were performed using JMP ver. 14.2.0 (SAS Institute Japan, Tokyo, Japan).

### Ethics

The study protocol was approved by the Clinical Research Ethics Review Committee in Saga University Hospital, and informed consent was obtained from all the subjects. This study was performed in accordance with the principles of the 1975 Declaration of Helsinki, revised in 2013.

## Results

### Patients’ characteristics

The patients’ characteristics are summarized in Table 1. The median age was 63 years and 64 (57%) patients were female. Diabetes and hypertension were diagnosed in 75 (66%) and 63 (56%) patients, respectively. The prevalence of mild, moderate, and severe pathological liver steatosis was 64.6%, 25.7%, and 9.7%, respectively. The median SCD was 19.9 mm.

**Table 1:** Patients’ characteristics.

**Clinical characteristics**	
Female, n (%)	64 (57)
Age, years*	63 (20–84)
Body mass index, kg/m^2^*	28.0 (18.8–38.5)
Diabetes, n (%)	75 (66)
Hypertension, n (%)	63 (56)
**Laboratory tests**	
Platelet count, × 10^3^/µL*	194 (59–637)
PT-INR	1.05 (0.91–1.49)
AST, U/L*	54 (15–259)
ALT, U/L*	53 (14–324)
ALP, U/L*	233 (106–818)
γGT, U/L*	61.5 (14–751)
T-BIL, mg/dL*	0.9 (0.4–2.2)
Total protein, g/dL*	7.1 (5.9–8.1)
Albumin, g/dL*	4.1 (3.2–5.0)
TC, mg/dL*	179 (109–275)
HDL-C, mg/dL*	46.5 (27–75)
LDL-C, mg/dL*	113 (42–198)
TG, mg/dL*	139 (38–513)
FPG, mg/dL*	107 (79–249)
T4C7s, ng/mL*	5.6 (2.6–14.0)
Ferritin, ng/dL*	245 (16–874)
**Pathological findings**	
Steatosis score 1/2/3, n	73/29/11
Lobular inflammation score 0/1/2/3, n	5/76/26/6
Ballooning score 0/1/2, n	46/39/28
Fibrosis stage 0/1/2/3/4, n	18/37/20/35/3
**Ultrasonographical findings**	
LSM, kPa*	8.8 (3.6–30.7)
CAP, dB/m*	283 (149–386)
SCD, mm*	19.9 (10.4–25.0)

*Data are shown as n, n (%), or median (range). Abbreviations: PT-INR, prothrombin time–international normalized ratio; AST, aspartate aminotransferase; ALT, alanine aminotransferase; ALP, alkaline phosphatase; γGT, γ-glutamyl transpeptidase; T-BIL, total bilirubin; TC, total cholesterol; HDL-C, high-density lipoprotein cholesterol; LDL-C, low-density lipoprotein cholesterol; TG, triglycerides; FPG, fasting plasma glucose; T4C7s, type IV collagen 7 s; LSM, liver stiffness measurement; CAP, controlled attenuation parameter; SCD, skin–capsular distance.

### Correlation between CAP and various parameters

**Figure 1:**
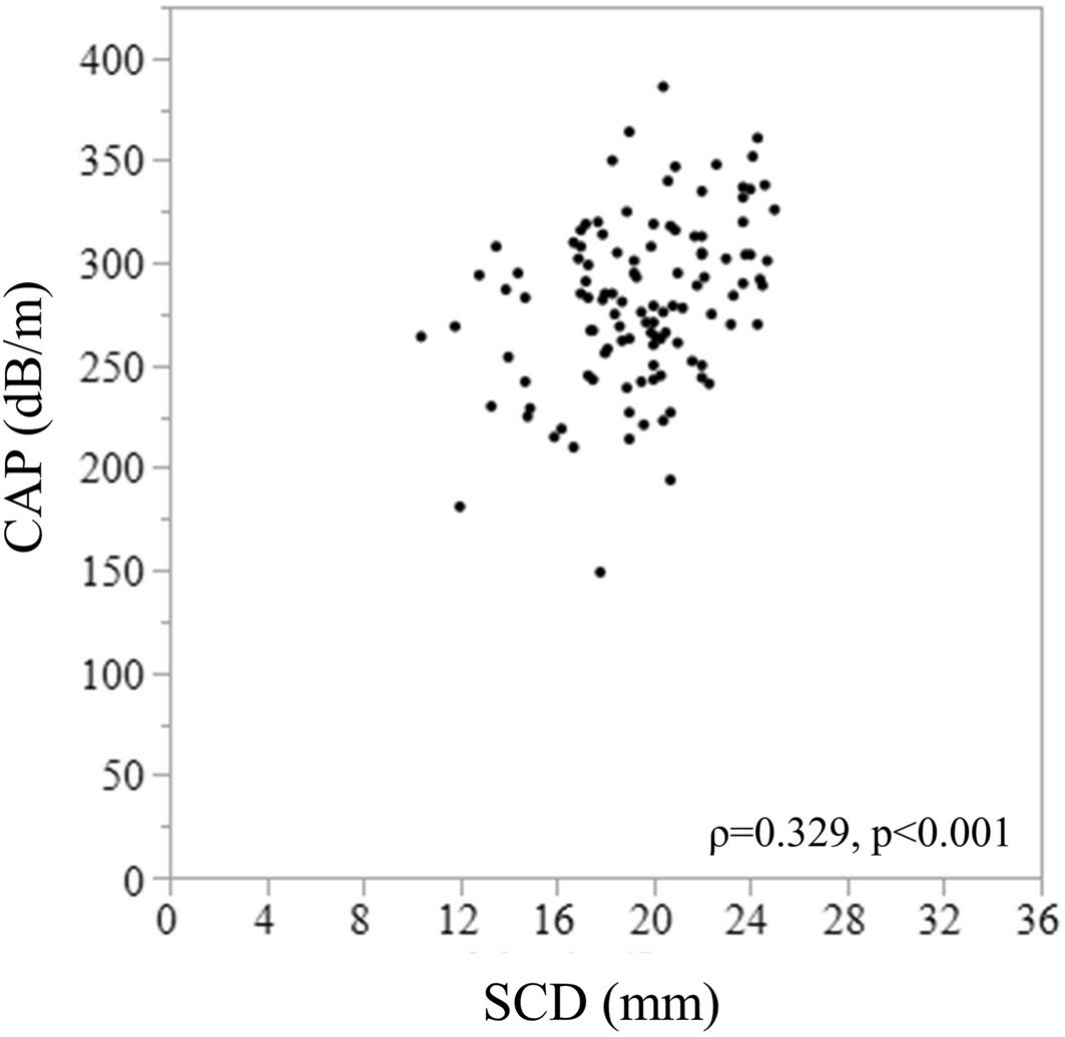
Correlation between CAP and SCD. CAP, controlled attenuation parameter; SCD, skin–capsular distance. Shaded error band represents 95% confidence interval.

The BMI did not show a significant correlation with the CAP; however, the albumin concentration showed a significant positive correlation with the CAP (ρ = 0.245, p = 0.009) (Table 2). Among all parameters, the SCD showed the most significant positive correlation with the CAP (ρ = 0.329, p < 0.001) (Table 2 and Fig. 1). Correlations between the CAP and pathological findings were also tested (Supplementary Table 1). The CAP increased as the steatosis score increased. The CAP decreased as the ballooning score increased, but there was no significant difference between the scores. The CAP significantly decreased in the patients with cirrhosis.

**Table 2:** Correlations of various parameters with CAP.

	ρ*	P value
Age	−0.100	0.292
Body mass index	0.171	0.067
Platelet count	0.106	0.263
PT-INR	−0.094	0.327
AST	−0.065	0.497
ALT	−0.008	0.937
ALP	−0.042	0.656
γGT	0.038	0.692
T-BIL	−0.008	0.931
Total protein	−0.040	0.677
Albumin	0.245	0.009
TC	0.069	0.470
HDL-C	−0.160	0.096
LDL-C	0.082	0.392
TG	0.097	0.308
FPG	0.151	0.111
T4C7s	−0.098	0.312
Ferritin	0.115	0.229
LSM	−0.022	0.815
SCD	0.329	< 0.001

PT-INR, prothrombin time–international normalized ratio; AST, aspartate aminotransferase; ALT, alanine aminotransferase; ALP, alkaline phosphatase; γGT, γ-glutamyl transpeptidase; T-BIL, total bilirubin; TC, total cholesterol; HDL-C, high-density lipoprotein cholesterol; LDL-C, low-density lipoprotein cholesterol; TG, triglycerides; FPG, fasting plasma glucose; T4C7s, type IV collagen 7 s; SCD, skin–capsular distance.

*Spearman’s correlation coefficient.

### Factors associated with CAP

Single and multiple regression analyses were performed to identify the factors associated with the CAP. Single regression analysis showed that sex (p = 0.027), BMI (p = 0.040), albumin (p = 0.002), pathological steatosis (p = 0.006), pathological ballooning (p = 0.011), and SCD (p < 0.001) were significantly associated with the CAP (Table 3). Multiple regression analysis demonstrated that the SCD and pathological steatosis were independently associated with the CAP in Model 1 (steatosis: β = 0.318, p < 0.001; SCD: β = 0.408, p < 0.001), Model 2 (steatosis: β = 0.334, p < 0.001; SCD: β = 0.474, p < 0.001), and Model 3 (steatosis: β = 0.306, p < 0.001; SCD: β = 0.415, p < 0.001) (Table 4). The albumin concentration was associated with the CAP in Model 3 (β = 0.227, p = 0.008). The BMI was not significant factors in any regression analysis models. According to the estimate in the multiple regression analysis, a 1-mm increase in the SCD resulted in a 5.26- to 6.11 dB/m increase in the CAP.

**Table 3:** Single regression analysis for factors associated with CAP.

	B*	SE	P value
Age	−0.328	0.282	0.248
Gender Male	−8.470	3.775	0.027
Body mass index	1.883	0.907	0.040
Platelet count	0.075	0.048	0.120
PT-INR	−36.407	45.660	0.427
AST	−0.042	0.104	0.686
ALT	0.017	0.077	0.826
ALP	−0.028	0.037	0.451
γGT	−0.001	0.034	0.966
T-BIL	−1.830	10.463	0.862
Total protein	−1.967	7.844	0.802
Albumin	32.724	10.309	0.002
TC	0.080	0.106	0.453
HDL-C	−0.643	0.339	0.060
LDL-C	0.159	0.121	0.194
TG	0.077	0.045	0.088
FPG	0.201	0.123	0.106
T4C7s	−2.354	1.812	0.197
Ferritin	0.019	0.019	0.327
Steatosis score	15.537	5.560	0.006
Lobular inflammation score	−6.974	6.001	0.248
Ballooning score	−12.061	4.684	0.011
Fibrosis stage	−4.444	3.328	0.185
LSM	−0.427	0.626	0.497
SCD	4.641	1.142	< 0.001

*Partial regression coefficient. Abbreviations: SE, standard error; SCD, skin–capsular distance; PT-INR, prothrombin time–international normalized ratio; AST, aspartate aminotransferase; ALT, alanine aminotransferase; ALP, alkaline phosphatase; γGT, γ-glutamyl transpeptidase; T-BIL, total bilirubin; TC, total cholesterol; HDL-C, high-density lipoprotein cholesterol; LDL-C, low-density lipoprotein cholesterol; TG, triglycerides; FPG, fasting plasma glucose; T4C7s, type IV collagen 7 s.

**Table 4:** Multiple regression analysis for factors associated with CAP.

	B*	β**	SE	P value
**Model 1**				
Steatosis score	19.268	0.318	5.135	< 0.001
SCD	5.260	0.408	1.093	< 0.001
**Model 2**				
Steatosis score	20.233	0.334	5.223	< 0.001
SCD	6.110	0.474	1.380	< 0.001
Body mass index	−1.041	−0.107	1.032	0.3151
**Model 3**				
Steatosis score	18.559	0.306	5.009	< 0.001
SCD	5.353	0.415	1.337	< 0.001
Body mass index	−0.680	−0.070	0.994	0.495
Gender, male	−3.922	−0.096	3.363	0.246
Albumin	25.770	0.227	9.526	0.008
Ballooning score	−4.807	−0.095	4.334	0.270

Multiple regression analysis of individual models. *Partial regression coefficient and **standard partial regression coefficient. Abbreviations: SE, standard error; SCD, skin–capsular distance.

### Adjusted CAP and its diagnostic performance

**Figure 2:**
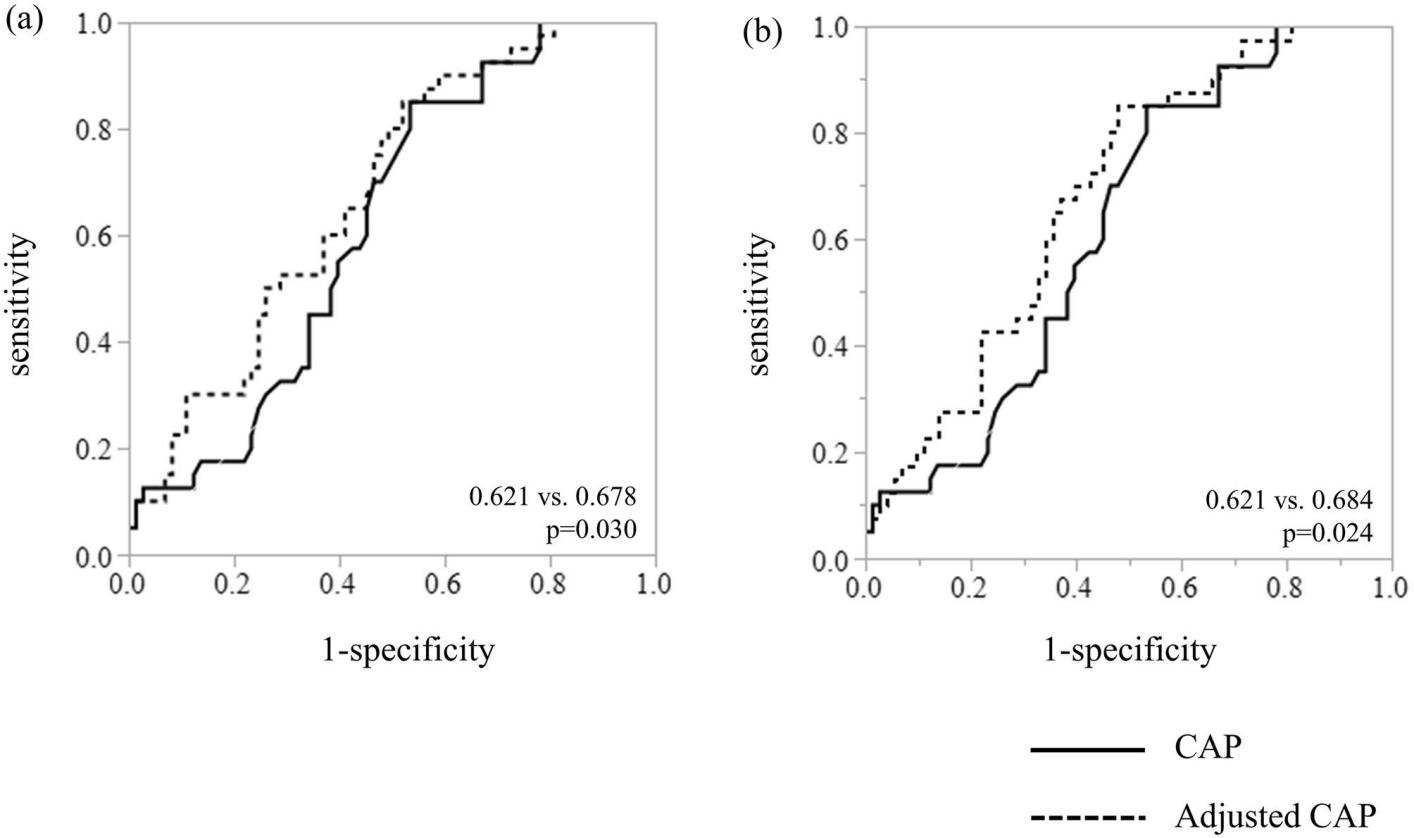
Comparison of the AUROC for the CAP (solid line) and (**a**) Model 1-adjusted CAP or (**b**) Model 3-adjusted CAP (dotted line) for detecting patients with a steatosis score ≥ 2. The p value was obtained by the DeLong test.

According to the partial regression coefficient in the multiple regression analysis of Model 1 and 3, we devised the following formulas to calculate the adjusted CAP: Model 1-adjusted CAP (dB/m) = CAP − (5.26 × SCD); Model 3-adjusted CAP (dB/m) = CAP − (5.35 × SCD) − (25.77 × serum albumin concentration). The AUROC of the Model 1-adjusted CAP for detecting patients with a steatosis score ≥ 2 was 0.678, which was significantly greater than the CAP without adjustment (0.621 vs. 0.678, p = 0.030) (Fig. 2a). According to Youden’s index, the cut-off value of the Model 1-adjusted CAP for the diagnosis of steatosis with a score of ≥ 2 was 165 dB/m with 85.0% sensitivity and 47.9% specificity, which was greater than that of the original CAP (Table 5). The positive predictive value and negative predictive value of the adjusted CAP were greater than those of the original CAP. Like the Model 1-adjusted CAP, the AUROC of the Model 3-adjusted CAP was also significantly greater than the CAP without adjustment (0.621 vs. 0.684, p = 0.024) (Fig. 2b). The cut-off value according to Youden’s index was 64 dB/m with 85.0% sensitivity and 52.1% specificity, which was greater than that of the original CAP (Table 5). There were no significant differences in the AUROC between the Model 1-adjusted CAP and the Model 3-adjusted CAP (0.678 vs. 0.684, p = 0.673).

**Table 5:** Cut-off values of original and modified CAP for detecting steatosis score of ≥ 2.

	CAP	Cut-off (dB/m)	Se	Sp	PPV	NPV
YI	Original	269	85.0	46.6	46.6	85.0
	Model 1-adjusted	165	85.0	47.9	47.2	85.4
	Model 3-adjusted	64	85.0	52.1	49.3	86.4
Se ≥ 90%	Original	258	90.0	32.9	42.4	85.7
	Model 1-adjusted	161	90.0	41.1	45.6	88.2
	Model 3-adjusted	48	90.0	34.2	42.9	86.2
Sp ≥ 90%	Original	335	12.5	90.4	41.7	65.3
	Model 1-adjusted	220	22.5	90.4	56.3	68.0
	Model 3-adjusted	113	20.0	90.4	53.3	67.3

All values are shown as percentages. Abbreviations: CAP, controlled attenuation parameter; YI, Youden’s index; Se, sensitivity; Sp, specificity.

## Discussion

The present study showed that the SCD was independently associated with the CAP as measured by the M probe of the FibroScan^®^ for the evaluation of liver steatosis in patients with NAFLD. The SCD was positively correlated with the CAP, suggesting that evaluation based on the CAP can result in overdiagnosis of liver steatosis in patients with NAFLD who have a greater SCD. Moreover, we generated a formula to calculate the adjusted CAP using the SCD, and the diagnostic performance of the CAP was improved. The diagnostic performance of the CAP varies among previous studies; the AUROC for the diagnosis of a steatosis score of ≥ 2 reported in individual studies ranges from 0.64 to 0.86 for the M probe^16^. The result of the present study was similar with these previous reports. Recent studies have shown that the diagnostic performance of the MRI-PDFF is superior to that of the CAP in detecting and grading liver steatosis in patients with NAFLD^35,36^. However, because of the high costs and long time required for the examination as well as the low availability of resources, it is difficult to measure the MRI-PDFF for screening and evaluation of disease progression in all patients with NAFLD, even in the hospital setting. Therefore, the diagnostic performance of the CAP must be improved for more accurate evaluation of steatosis.

The BMI has been considered to be a factor associated with the CAP in various chronic liver diseases, including NAFLD^11,16,23–28^. In the present study, however, the independent association between the BMI and CAP was negated when the SCD was included as an explanatory variable for the multiple regression analysis. Furthermore, the SCD showed a more significant correlation with the CAP than BMI. The present study raises the following question: Why is the SCD, not the BMI, independently associated with the CAP? We measured the SCD in the right intercostal region and defined it as the total distance of the tissue layers between the skin and liver surface. Because these tissue layers mainly comprise subcutaneous fat, the SCD can be used to represent the amount of subcutaneous fat. Adiposity is one of the major findings of obesity. Subcutaneous fat accumulation, as well as visceral fat accumulation, is associated with the pathogenesis of NALFD^37–41^. The distance of the abdominal subcutaneous fat measured by ultrasound is reportedly associated with the severity of liver steatosis in adults with NAFLD and is a more accurate predictor of severe liver steatosis than is the BMI^40^. Similarly, in obese children, the abdominal subcutaneous fat thickness measured by ultrasound is positively correlated with the severity of liver steatosis^41^. Like the visceral adipose tissue, the deep layer of the subcutaneous adipose tissue shows higher expression of inflammatory genes than does the superficial subcutaneous adipose tissue, and such gene expression is correlated with hepatic steatosis and fibrosis in patients with NAFLD^42^. These findings suggest that the subcutaneous fat is significantly associated with liver steatosis and that the SCD, including the subcutaneous fat thickness, might be more closely related to liver steatosis than the BMI, which is always affected by the lean mass including the skeletal muscle and bone.

Because the CAP is calculated by the attenuation of the ultrasound pulse, the SCD can directly affect the CAP. The FibroScan^®^ manufacturer, Echosens, recommends that the FibroScan^®^ probe selection be based on the SCD; specifically, the M probe should be used for patients with an SCD of ≤ 25 mm, and the XL probe for should be used for those with an SCD of > 25 mm and ≤ 35 mm. In 2015, Shen et al.^43^ first reported that the SCD affected the CAP in patients with NAFLD and chronic hepatitis B (n = 381). At that time, the CAP was available only for the M probe, and its diagnostic accuracy and association with the SCD had been tested using only the M probe. The association of the SCD with the CAP independent of pathological steatosis and CAP was overestimated in patients with an SCD of ≥ 25 mm. Today, the CAP is available for both the M probe and XL probe, and CAP measurement using the M probe in patients with an SCD of ≥ 25 mm is outside the indication for the M probe; such patients should be examined using the XL probe. Interestingly, the SCD affected the CAP even in patients whose SCD was within the indications of the individual probes. Our data suggest for the first time that the effect of the SCD on the CAP is not determined by a threshold such as the upper range of the probe indication of the SCD but is consecutively observed within the indication of the SCD in patients with NAFLD. More interestingly, the standard partial regression coefficient of SCD is higher than that of steatosis in any multiple regression analysis models, which means that the effect of SCD on CAP is greater than that of steatosis. Therefore, it makes sense that the diagnostic performance of CAP improves by using the adjusted CAP.

In the present study, the effect of the SCD on patients with an SCD exceeding the probe indication remains unclear; this can be considered a study limitation. According to a previous report, optimization of the cut-off value might be needed for CAP measurement in patients with an SCD greater than the probe indication, at least for the M probe measurement^43^. The adjusted CAP in the present study should be validated in further multicenter studies that include patients with an SCD exceeding the probe indication. Additionally, the adjusted CAP should be tested in a study cohort that includes subjects without NAFLD (healthy subjects) to determine its detecting capability in the primary care setting and during health check-ups. The effect of inter- and intra-operator error on the CAP measurement and the diagnostic performance of the adjusted CAP remains unclear in the present study. Recently, high inter- and intra-operator reproducibility of the FibroScan was demonstrated^44^. Further studies are required to test the effects of inter- and intra-operator error on the CAP and the adjusted CAP.

In conclusion, the SCD is positively correlated with the CAP and is associated with the CAP independent of steatosis. Adjustment of the CAP using the SCD improves the diagnostic performance of the CAP in patients with NAFLD.

## Supplementary Information

Supplementary Information.
