# Supplementary material for: Effect of skin–capsular distance on controlled attenuation parameter for diagnosing liver steatosis in patients with nonalcoholic fatty liver disease
Source: Sci Rep. 2021 Aug 2;11:15641. doi: 10.1038/s41598-021-94970-3 (PMC8329228; doi:10.1038/s41598-021-94970-3)

**Supplementary Table 1. Median values of CAP and correlation with histologic findings**

|  | n | CAP (dB/m) | ρ | P value |
| --- | --- | --- | --- | --- |
| Steatosis score |  |  | 0.2181 | 0.020 |
| 1 | 73 | 275 (149-361) |  |  |
| 2 | 29 | 285 (242-364) |  |  |
| 3 | 11 | 305 (243-386) |  |  |
| Inflammation score |  |  | -0.0610 | 0.521 |
| 0 | 5 | 252 (221-340) |  |  |
| 1 | 76 | 286 (194-386) |  |  |
| 2 | 26 | 281 (149-364) |  |  |
| 3 | 6 | 249.5 (181-290) |  |  |
| Ballooning score |  |  | -0.2152 | 0.022 |
| 0 | 46 | 293 (210-352) |  |  |
| 1 | 39 | 276 (219-386) |  |  |
| 2 | 28 | 274 (149-361) |  |  |
| Fibrosis stage |  |  | -0.1038 | 0.274 |
| 0 | 18 | 291 (221-352) |  |  |
| 1 | 37 | 289 (210-361) |  |  |
| 2 | 20 | 272 (149-326) |  |  |
| 3 | 35 | 285 (194-386) |  |  |
| 4 | 3 | 219 (181-244) |  |  |

Correlations were tested using Spearman’s correlation coefficient. CAP is shown as median (range).

Abbreviation: CAP, controlled attenuation parameter

**Supplementary Figure 1. The skin–capsular distance (SCD). The distance between the skin surface and the liver capsule (↔).**


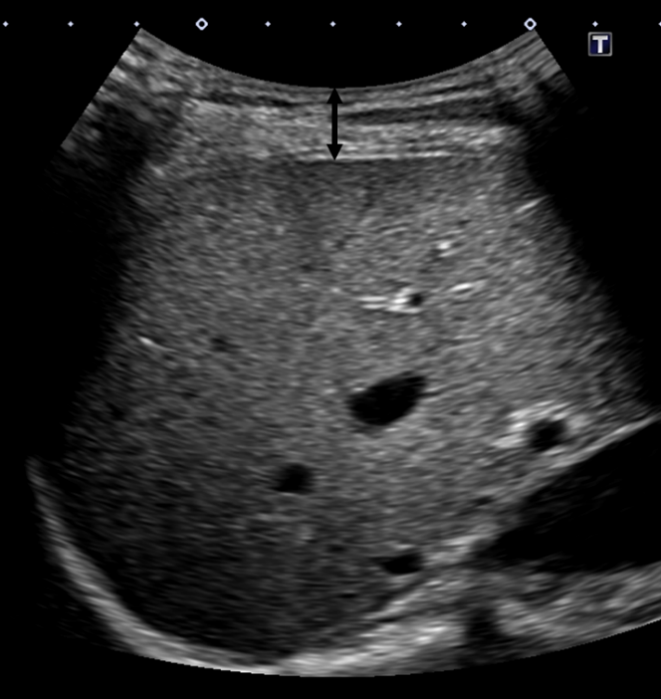

Supplement: Supplementary file 1 — Supplementary Information. [file 41598_2021_94970_MOESM1_ESM.docx]
